# Gastric Cardia Adenocarcinoma with Metastasis to the Scalp: A Case Report

**DOI:** 10.7759/cureus.6781

**Published:** 2020-01-27

**Authors:** Sanjay V Menghani, Alexandra Barbosa, Paul Sagerman, Matthew W Beal, Aaron Scott

**Affiliations:** 1 Internal Medicine, University of Arizona College of Medicine, Tucson, USA; 2 Pathology, Pathology Biomedical Consulting, Tucson, USA; 3 Dermatology, University of Arizona College of Medicine, Tucson, USA; 4 Oncology, University of Arizona Cancer Center, Tucson, USA

**Keywords:** gastric cardia adenocarcinoma, pembrolizumab, scalp metastasis, cutaneous metastasis, pd-l1

## Abstract

Cutaneous metastasis is a rare manifestation of advanced gastrointestinal (GI) cancers. Gastric adenocarcinoma rarely presents with cutaneous metastasis, as cutaneous manifestations occur in less than 1% of upper GI tract malignancies. Here, we present the case of a patient with advanced gastric cardia adenocarcinoma with metastasis to the right occipital region of the scalp. Following shave biopsy, the immunohistochemistry (IHC) and molecular profile of the scalp lesion were analyzed, both of which confirmed metastasis and guided the treatment approach. The lesion demonstrated programmed death ligand-1 (PD-L1), an immune checkpoint protein, positivity by IHC, which led to the recommendation for treatment with immunotherapy as per the National Comprehensive Cancer Network (NCCN) guidelines. Clinicians should conduct dermatologic examinations in patients with a history of gastric cancer or who are currently undergoing chemotherapy for gastric cancer in order to monitor for disease progression or metastatic lesions. The aim of this report is to increase awareness of scalp metastasis as an indicator of advanced internal visceral carcinoma for earlier diagnosis and improved management of the condition.

## Introduction

Cutaneous metastasis is a rare manifestation of advanced gastrointestinal (GI) cancers. While other tumor types such as lung, breast, and prostate cancers have well-documented cases of metastasis to the skin and scalp, gastric adenocarcinomas rarely display cutaneous metastasis, and it accounts for less than 1% of upper GI tract malignancies [1-3]. Patients with advanced gastric cancer (defined as locally advanced or metastatic) have a dismal prognosis with a median survival of 10-12 months [4]. While the liver, peritoneum, lung, and bone are the common sites of metastases for gastric adenocarcinoma, here we describe a case where a patient presented to the clinic with a growing scalp lesion that was subsequently biopsied positive for metastatic gastric cancer.

## Case presentation

A 69-year old male with a past medical history of osteoarthritis and sarcoidosis presented to the GI oncology clinic after a referral from gastroenterology. He complained of difficulty in swallowing and unintended weight loss of 18 pounds over two months. He was obese with a BMI of 32 with no history of gastroesophageal reflux disease (GERD) or no known history of *Helicobacter pylori* (*H. pylori) *infection. His family history was positive for kidney cancer in his father and stomach cancer in a maternal aunt. Diagnostic and staging evaluation with endoscopic ultrasound (EUS), endoscopic biopsy, and PET/CT scan confirmed a Siewert type III GEJ, cT3N0Mx, moderately differentiated adenocarcinoma. *H. pylori *immunohistochemical ​(​IHC) testing was negative. Upon multidisciplinary review with medical oncology, surgical oncology, radiation oncology, and gastroenterology, the patient was offered perioperative chemotherapy as standard of care (SOC) per NCCN guidelines [5,6]. However, the patient reported that he had “connections” for “better treatment options” through non-disclosed clinics outside of the US and was lost to follow-up.

**Figure 1:**
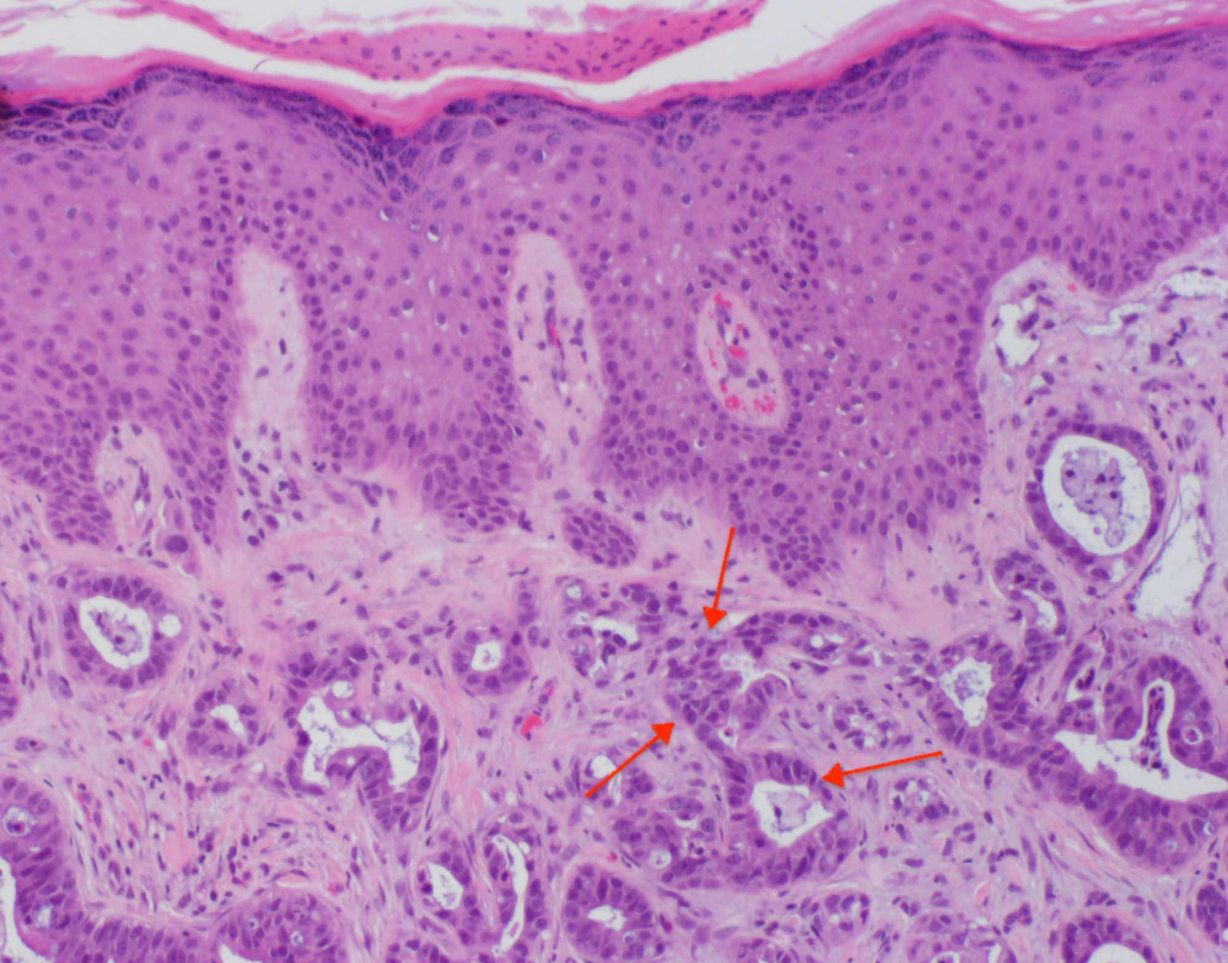
Right occipital scalp lesion dermatopathology H&E stain showing well-formed glandular structures present with focal lymphatic invasion; red arrows indicate representative focal lymphatic invasion and glandular structures (original magnification x200) H&E: hematoxylin and eosin

**Figure 2:**
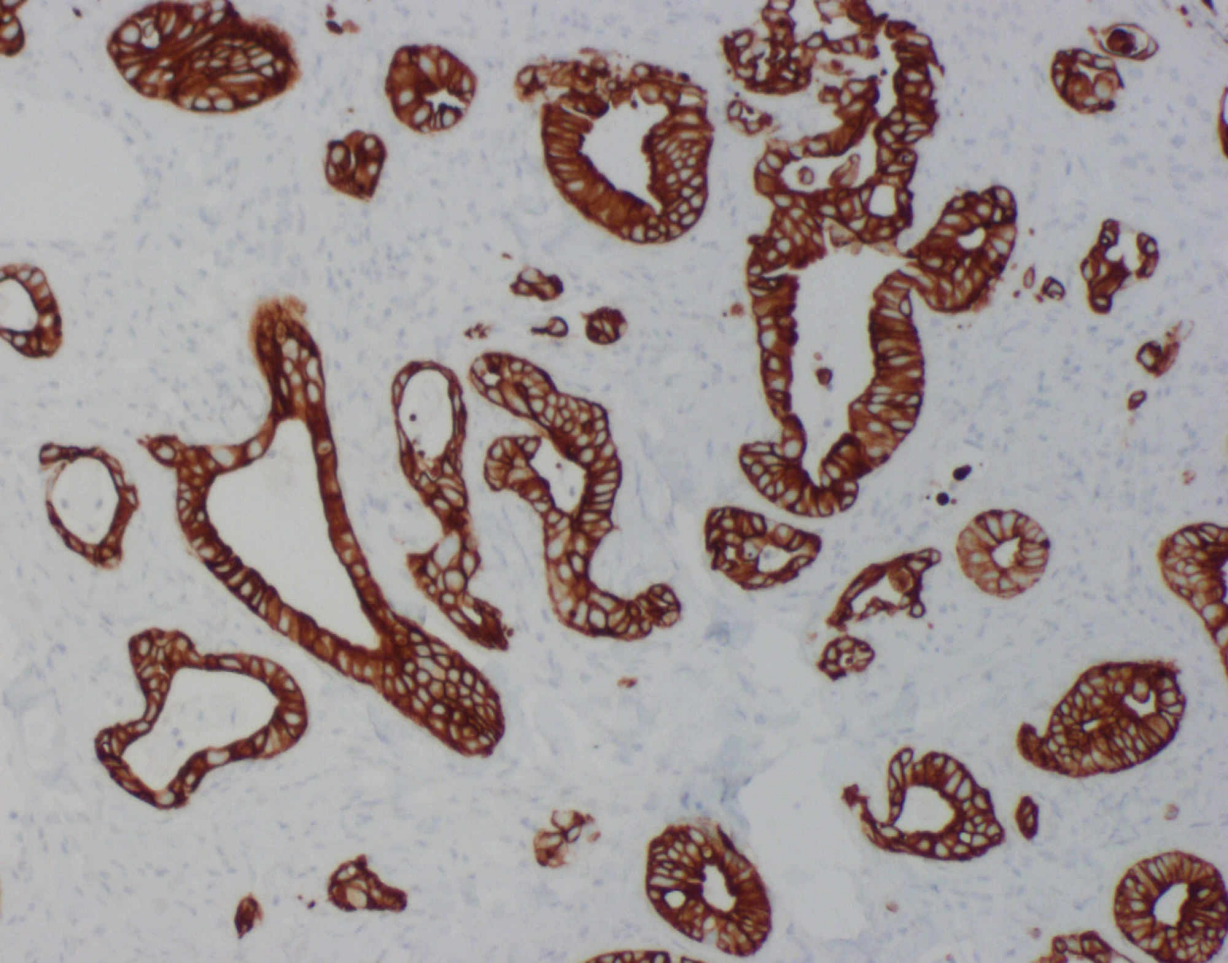
Right occipital scalp lesion immunohistochemistry showing positivity for cytokeratin 20 (original magnification x100)

**Figure 3:**
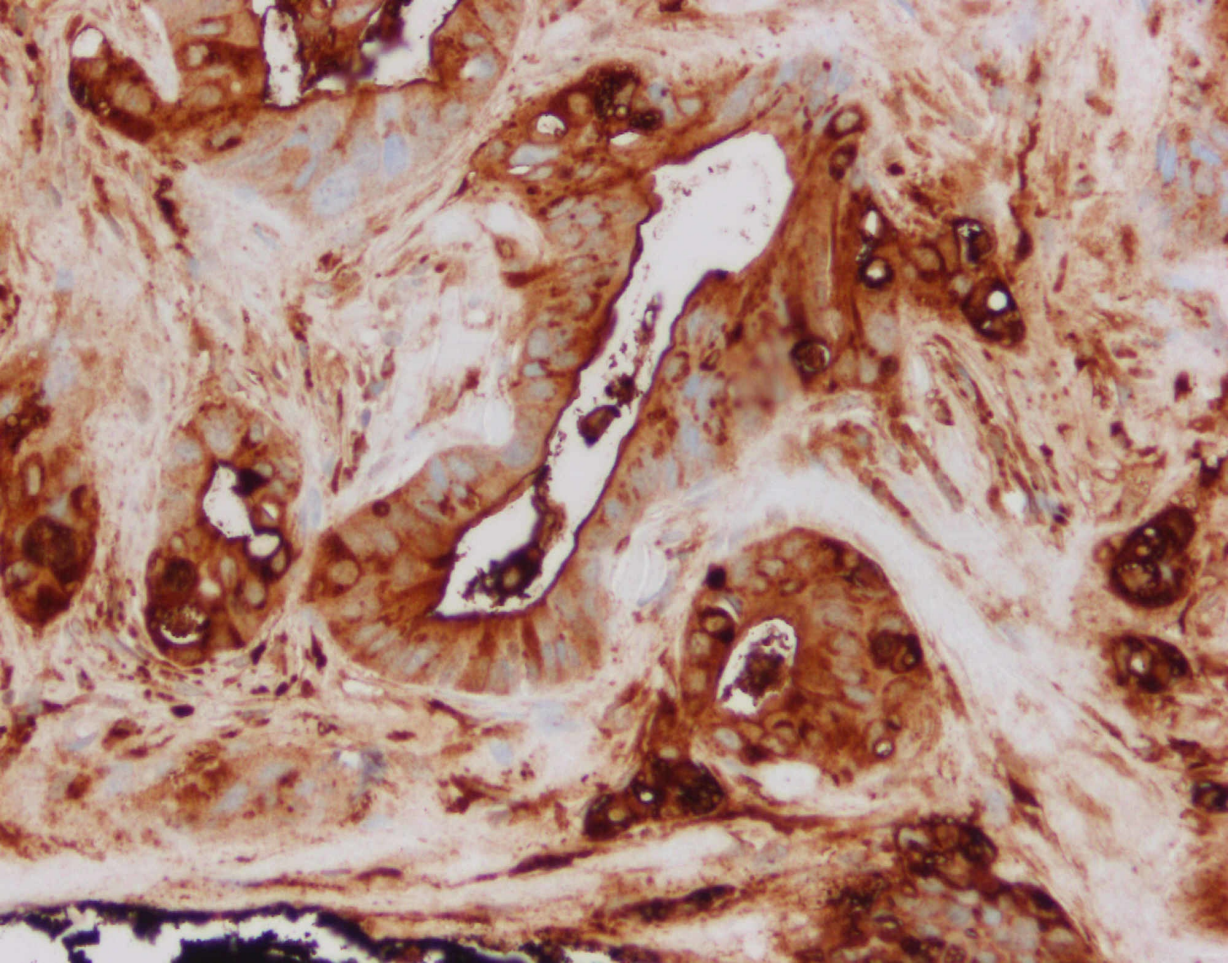
Right occipital scalp lesion immunohistochemistry showing positivity for CEA (original magnification x200) CEA: carcinoembryonic antigen

**Figure 4:**
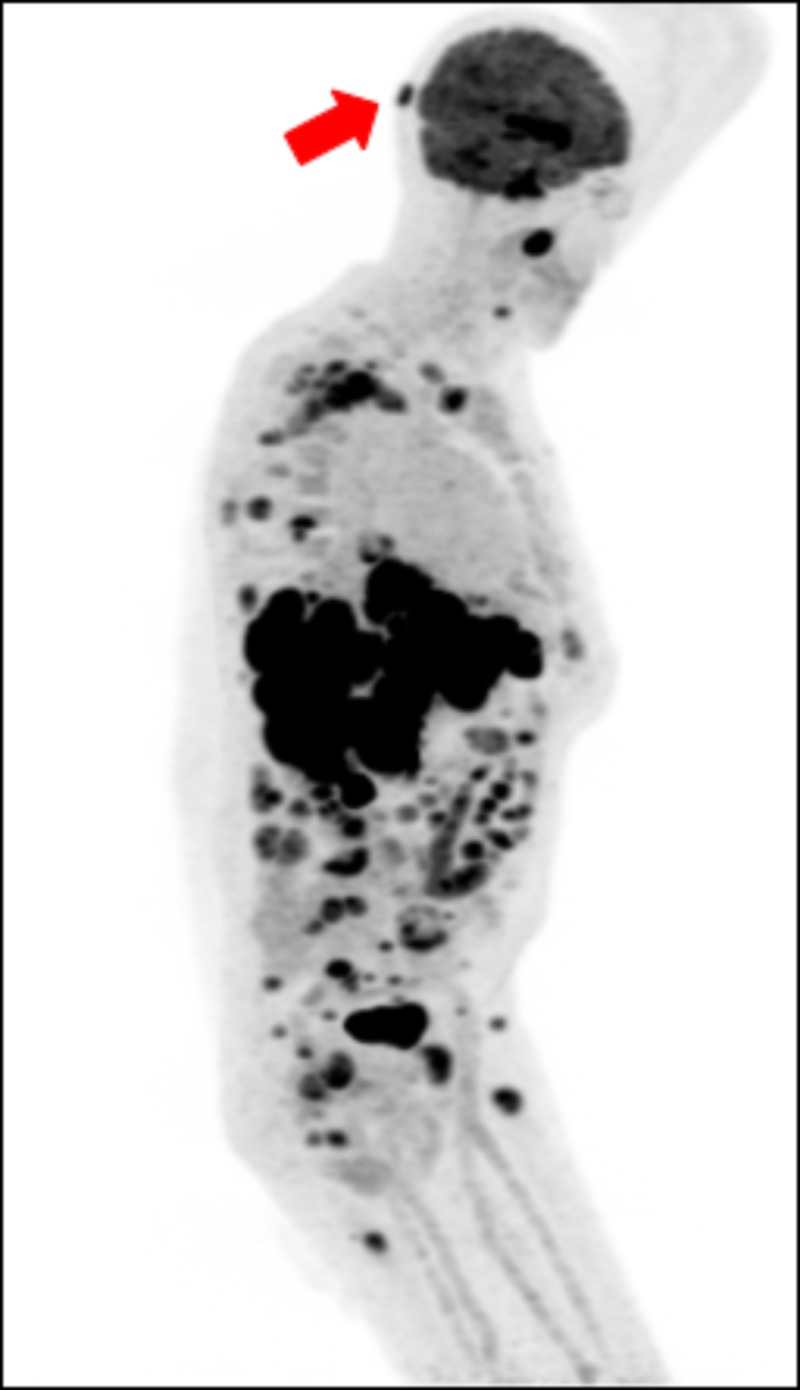
Restaging PET/CT image of the patient Restaging image obtained with evidence of right occipital metastasis; sagittal view presented with red arrow indicating occipital lesion PET; positron emission tomography; CT: computed tomography

Approximately 20 months post-initial diagnosis, the patient returned to the clinic reporting that he had received a variety of non-SOC “curative treatments” in Mexico and China. He noted that he had a scalp lesion that continued to grow and was bothersome, which he attributed to bedsore. Upon dermatologic exam, a friable plaque with a yellow scale and crusts was located in the right occipital region of the scalp. The scalp overall was erythematous and tender to palpation. Dermatopathology review of the biopsied scalp lesion showed well-formed glandular structures present through the dermal portion of the biopsy with focal lymphatic invasion (Figure 1). The lesion was positive for expression of cytokeratin 7, cytokeratin 20 (Figure 2), and carcinoembryonic antigen (CEA) via IHC (Figure 3). Stains for caudal type homeobox 2 (CDX2) and special AT-rich sequence-binding protein 2 (SATB2) were not done. The lesion was negative for expression of P63, thyroid transcription factor 1 (TTF1), and prostate-specific antigen (PSA). The final pathologic report concluded that this was metastatic adenocarcinoma consistent with his history of gastric cancer. This prompted a restaging of PET/CT, which showed diffuse fluorodeoxyglucose (FDG)-avid metastatic disease including liver, skeleton, and occipital lesions (Figure 4). The biopsy specimen of the scalp was sent for CARIS Molecular Intelligence (Caris Life Sciences, Inc., Dallas, TX) profiling, which revealed the lesion to be positive for programmed death ligand-1 (PD-L1) upregulation by IHC.

The molecular information derived from the scalp lesion helped guide management as immunotherapy has been FDA-approved for the treatment of PD-L1-positive metastatic gastric and esophageal cancers in the subsequent line setting. Expression of PD-L1 has been shown to be a prognostic indicator for immunotherapy in metastatic gastric adenocarcinoma [7]. Objective responses to immune checkpoint inhibitor therapy have been shown, which led to the FDA approval of pembrolizumab, an anti-PD-1 immune checkpoint inhibitor, as a ≥3rd-line treatment option for PD-L1 expressing with a combined positive score (CPS) of ≥1 in metastatic gastric cancer [8-10]. As our patient had progressed on multiple chemotherapy regimens and since his molecular pathology demonstrated PD-L1 positivity with CPS 1, he was started on pembrolizumab monotherapy. Unfortunately, due to extensive metastatic disease, the patient passed away several weeks after the initiation of pembrolizumab.

## Discussion

Gastric adenocarcinomas rarely display cutaneous metastasis, but case reports of this have been reported over the years. Previously reported cases of cutaneous metastatic manifestations of gastric adenocarcinoma have described symptoms reported 3-10 years after the primary diagnosis [11]. Cutaneous metastases have been described as part of the natural history of disease progression in individual patients [11]. A brief literature review of PubMed articles indexed for MEDLINE yielded 10 relevant case reports with similarities to our patient, but with noted differences. Several cases, including those discussed by Lifshitz et al., Woo et al., and Cho et al., describe scalp or skin metastasis as a recurrence of gastric cancer [12-14]. The report by Sakaki et al. from 1979 describes scalp and dural lesions in the right parietal region with gastric adenocarcinoma found at autopsy [15]. Several case reports describe alopecia neoplastica as a paraneoplastic sequela of gastric adenocarcinoma [16,17]. The case report and literature review by Du et al. do not describe the IHC or molecular profile of the lesion, focusing instead on radiology- and pathology-based evidence [18]. A further review of the relevant results showed no results utilizing the IHC profile and molecular profiling of the metastatic lesion to guide the treatment approach for both primary and metastatic lesions.

Overall, our patient had a relatively poor prognosis from his initial PET/CT. Patients with advanced gastric cancer (defined as locally advanced or metastatic) have a median survival of 10-12 months [4]. The National Cancer Institute Surveillance, Epidemiology, and End Results Program (SEER) database reports an overall 5-year survival rate from gastric cancer of 31.5%. Patients with a distant disease (defined as having metastasized) have a 5-year relative survival rate of 5.3%. In gastric cancer treatment, the use of immunotherapy has been a growing trend. For patients enrolled in Phase 2 KEYNOTE-180 study with gastric adenocarcinoma that progressed over two treatment regimens and with PD-L1-positive tumors, there was a 13.8% objective response rate [8,9]. The main benefits of anti-PD-1 immunotherapeutic agents are the manageable safety profile and promising antitumor activity in this setting [10]. For our patient, cutaneous metastasis was a distinct sign of disease progression and the use of pembrolizumab was palliative. As shown by the PET/CT image, there was evidence of diffuse FDG-avid metastatic disease affecting the liver, skeleton, and occipital region. The liver and skeleton are locations where the metastatic disease is commonly seen; however, the occipital lesion is uncommon in these organs [3].

## Conclusions

We reported a case of a patient with cutaneous scalp metastasis from a primary gastric cardia adenocarcinoma. Cutaneous metastasis is a rare but clinically accepted manifestation of advanced internal visceral carcinoma such as gastric adenocarcinoma. The scalp lesion IHC profile (cytokeratin 7, cytokeratin 20, and CEA positivity) and molecular profiling (PD-L1 positivity) were used to guide the treatment approach for both the primary and metastatic lesions. We encourage the clinicians to perform a thorough dermatologic examination in patients with a history of gastric cancer, especially in the setting of new dermatologic symptoms/findings during treatment and/or surveillance. We also aim to increase awareness regarding scalp metastasis as an indicator of advanced internal visceral carcinoma, which would help in earlier diagnosis and improved management of patients.
